# Selective PEGylation of Parylene-C/SiO_2_ Substrates for Improved Astrocyte Cell Patterning

**DOI:** 10.1038/s41598-018-21135-0

**Published:** 2018-02-09

**Authors:** B. J. Raos, C. S. Doyle, M. C. Simpson, E. S. Graham, C. P. Unsworth

**Affiliations:** 1 Department of Engineering Science, The University of Auckland, Private Bag 92019, Auckland, 1142 New Zealand; 2 Department of Chemical and Materials Engineering,, The University of Auckland, Private Bag 92019,, Auckland, 1142 New Zealand; 3 Department of Chemistry, The University of Auckland, Private Bag 92019, Auckland, 1142 New Zealand; 4 Department of Physics, The University of Auckland, Private Bag 92019, Auckland, 1142 New Zealand; 5 The Photon Factory, The University of Auckland, Private Bag 92019, Auckland, 1142 New Zealand; 6 The MacDiarmid Institute for Advanced Materials and Nanotechnology, Auckland, 1142 New Zealand; 7 The Dodd Walls Centre for Photonic and Quantum Technologies, Auckland, 1142 New Zealand; 8 Department of Pharmacology and Centre for Brain Research, School of Medical Sciences, Faculty of Medical and Health Sciences, The University of Auckland, Private Bag 92019, Auckland, 1142 New Zealand

## Abstract

Controlling the spatial distribution of glia and neurons in *in vitro* culture offers the opportunity to study how cellular interactions contribute to large scale network behaviour. A recently developed approach to cell-patterning uses differential adsorption of animal-serum protein on parylene-C and SiO_2_ surfaces to enable patterning of neurons and glia. Serum, however, is typically poorly defined and generates reproducibility challenges. Alternative activation methods are highly desirable to enable patterning without relying on animal serum. We take advantage of the innate contrasting surface chemistries of parylene-C and SiO_2_ to enable selective bonding of polyethylene glycol SiO_2_ surfaces, i.e. PEGylation, rendering them almost completely repulsive to cell adhesion. As the reagents used in the PEGylation protocol are chemically defined, the reproducibility and batch-to-batch variability complications associated with the used of animal serum are avoided. We report that PEGylated parylene-C/SiO_2_ substrates achieve a contrast in astrocyte density of 65:1 whereas the standard serum-immersion protocol results in a contrast of 5.6:1. Furthermore, single-cell isolation was significantly improved on PEGylated substrates when astrocytes were grown on close-proximity parylene-C nodes, whereas isolation was limited on serum-activated substrates due tolerance for cell adhesion on serum-adsorbed SiO_2_ surfaces.

## Introduction

Patterned cultures offer the opportunity to study cell communication at the cellular and network level. Of particular interest to the neuroscience community, the precise placement of both neurons and glia enables study of how the behaviour of single cells contribute larger scale network behaviour.

One cell-patterning technique that holds promise is the parylene-C/SiO_2_ platform developed by Delivopoulos *et al*.^1^. The platform utilises the biocompatible polymer parylene-C deposited onto a SiO_2_ background to create surfaces that are attractive and repulsive to cell adhesion, respectively. While a multitude of cell-patterning techniques are available^2^, the parylene-C/SiO_2_ platform is attractive for neuroscience research as it is easily integrated into multi-electrode arrays. Parylene-C layers as thin as 10 nm have successfully been used to pattern astrocytes, providing an alternative biocompatible insulating material for neural micro-electrodes^3^. Furthermore, parylene-C/SiO_2_ substrates can be manufactured by two complementary techniques: photolithography, which is suitable for mass production, and laser ablation, which provides a means to rapidly prototype pattern designs.

Delivopoulos *et al*. first used the parylene-C/SiO_2_ platform to guide the growth of murine neurons and glia^1^, confining their cell bodies to thin strips of parylene. Unsworth *et al*. subsequently used the platform to isolate single astrocytes on nodes of parylene^4^. In both these works, however, the glia displayed a limited tendency to grow into normally cell-repulsive SiO_2_ regions.

Cell patterning relies on contrasts in the ability of materials to support cell adhesion, typically achieved by surface modifications to make a substrate more or less attractive to cell adhesion. Cell adhesion to surfaces is controlled by a multitude of factors, including surface wettability^5^, topography^6^, charge^7^, and the surfaces mechanical properties^8^. The parylene-C/SiO_2_ platform achieves contrast in cell adhesion by incubating the parylene-C/SiO_2_ substrates in serum prior to cell culture. Delivopoulos *et al*. and Hughes *et al*. hypothesize that cell patterning is the result of the combinatorial effects resulting from the adsorption of specific serum proteins and the proteins conformation once adsorbed^1,9,10^.

While the original work of Delivopoulos *et al*.^1^ used equine serum (ES) to activate parylene-C/SiO_2_ substrates, Unsworth *et al*.^4^ investigated the effects of alternative serum sources, specifically, human serum (HS) and foetal bovine serum (FBS). Unsworth *et al*. demonstrated that FBS resulted in superior conformity to parylene regions compared to equivalent substrates immersed in ES and HS. Qualitatively, FBS immersed substrates achieved sharp cellular contrast on the parylene whereas other serum types produced a blotchy patterning of the neurons which spanned across the parylene and onto the SiO_2_ substrate.

A commonly acknowledged drawback of the use of serum in cell culture is the variability that occurs between batches, with for example the total protein content varying from 3.2 to 7 g/100 mL^11^. When the quality of patterning relies on the protein content of the serum it is undesirable to rely on sources of protein that are not consistent from batch-to-batch.

Hughes *et al*.^9^ attempted to circumvent the use of serum through the use of rationalized protein activation solutions, consisting of defined concentrations of the serum proteins. Hughes *et al*. reported that while cell attachment to parylene-C was achieved using pure solutions of the vitronectin, fibronectin, laminin, or collagen, that patterning was undermined by significant cell attachment to SiO_2_. Additionally, cell adhesion to parylene-C was 5 times poorer compared to serum-activated substrates. Delivopoulos *et al*.^10^ subsequently characterized the structural properties of fibronectin and serum albumin adsorbed to parylene-C and SiO_2_ surfaces. Significantly, Delivopoulos *et al*. reported that when fibronectin and albumin were co-incubated at the 1:100 ratio found in serum, fibronectin preferentially adsorbed to silicon oxide, while albumin preferentially adsorbed to parylene-C, despite the presence of a high concentration of albumin in the bulk solution. Though the work of Delivopoulos *et al*. and Hughes *et al*. has sought to mitigate the complications associated with the use of serum, a complete description of protein adsorption to parylene-C and SiO_2_ surfaces has not been elucidated.

Furthermore, a complicating factor when fabricating parylene-C/SiO_2_ substrates with the rapid-prototyping laser ablation technique is that sub-ablation-threshold laser machining induces nanoscale surface roughness on the SiO_2_ surface. Raos *et al*. demonstrated that the fidelity of glial patterning was dependent on the laser ablation parameters and that patterning was reduced compared to the standard photolithographic technique, which renders the SiO_2_ surface smoother^12^. While laser induced surface topography changes have been investigated as a method to pattern cultures of neural cells^13^ it is difficult to produce surfaces smooth enough to limit cell attachment using laser ablation.

In this work, we propose a simple alternative activation mechanism for the parylene-C/SiO_2_ cell-patterning platform through selective PEGylation of SiO_2_ regions. Polyethylene glycol (PEG) is a synthetic polymer that has been extensively studied for its antifouling properties. The ability of PEG films to resist protein adsorption has previously been used to facilitate cell patterning in a variety of contexts^14–16^. PEG is often bound to surfaces through a silanization reaction, whereby a functional organosilane reacts, through a condensation reaction, with free hydroxyl functional groups to covalently bond the silane molecule to the surface. The free functional group on the silane can then be bonded to PEG through complementary functionality on the PEG molecule. As parylene-C lacks free hydroxyl functional groups, the parylene-C/SiO_2_ platform intrinsically contains the relevant surface chemistries that enable selective PEGylation, without the need to resort to additional photolithographic steps.

Our motivations for this work are two-fold, first we aim to remove the requirement to activate parylene-C/SiO_2_ samples through serum adsorption by selectively bonding PEG to the SiO_2_ surfaces and second, we aim to improve the fidelity of glial cell patterning on the parylene-C/SiO_2_ through increased control of the astrocytic cytoplasm.

## Results

### Glial Cell-Patterning on PEGylated Parylene-C/SiO2 Substrates

**Figure 1:**
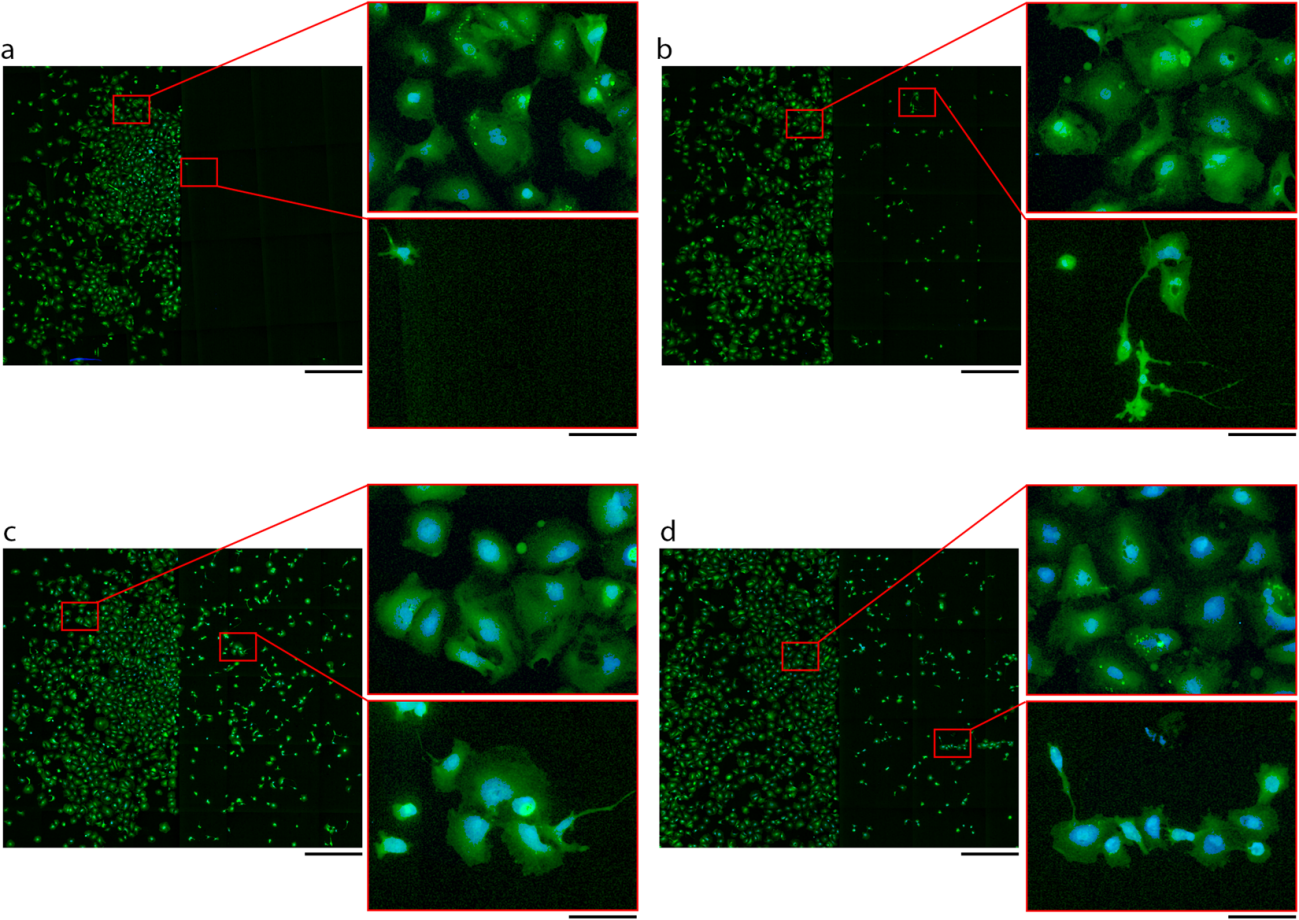
Characteristic images of hNT astrocytes cultured for 72 hours on PEGylated SiO_2_/parylene-C substrates and controls. Astrocyte cytoplasms were labelled with CMFDA (green) and the nuclei labelled with Hoechst 33258 (blue). Parylene-C/SiO_2_ substrates were treated with, (**a**) the PEGylation protocol, (**b**) the serum-immersion protocol, (**c**) the PEGylation protocol without APTES, and (**d**) the PEGylation protocol without m-PEG-SVA. Scale bars represent 1000 µm and 150 µm for the overview and cutout images, respectively. Images were modified by adjusting the contrast only.

**Figure 2:**
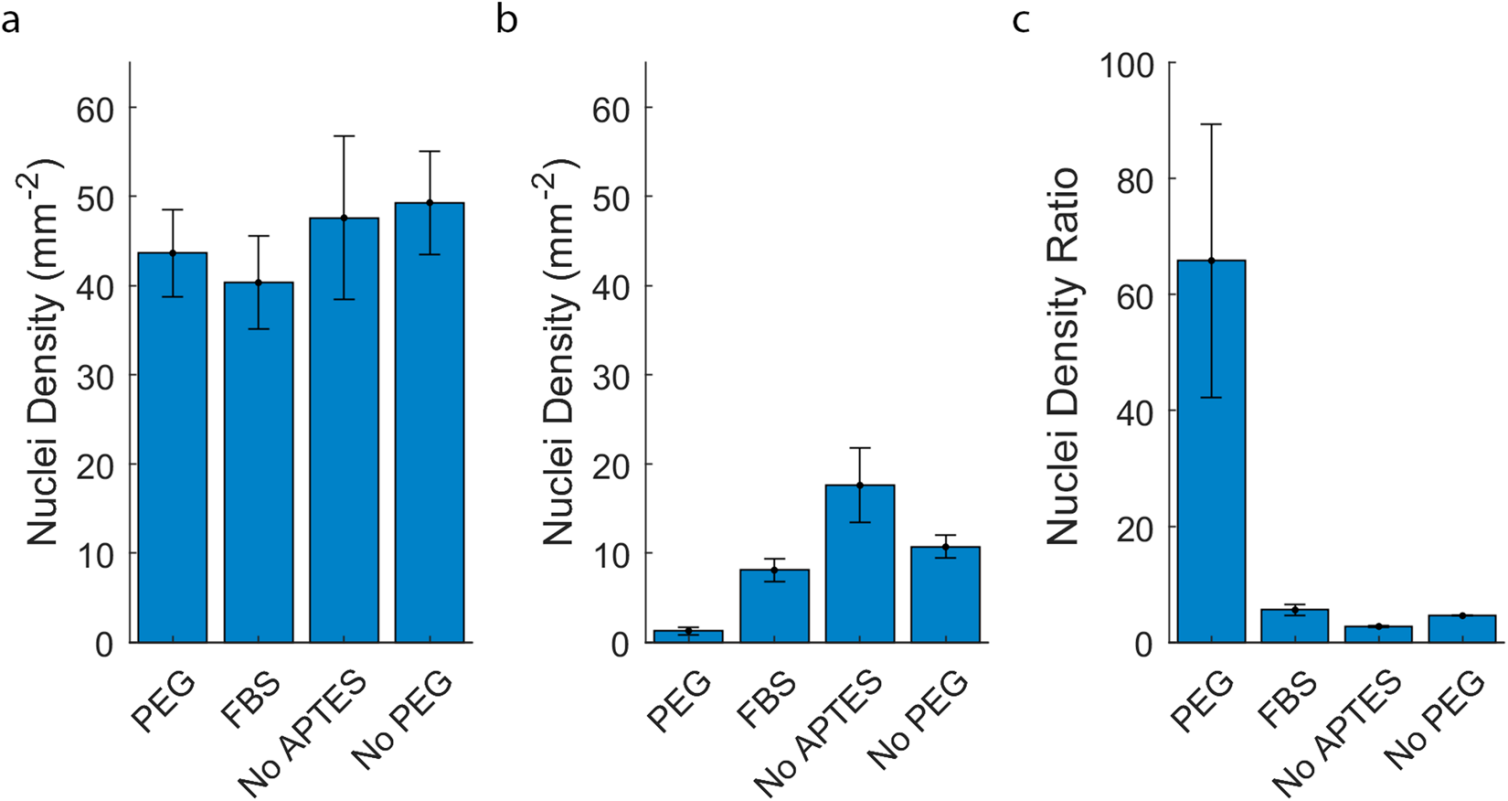
Quantification of cell patterning on PEGylated SiO_2_/parylene-C samples and controls. (**a**) Mean nuclei density on parylene-C surfaces. (**b**) Mean nuclei density on SiO_2_ surfaces. (**c**) Mean ratio of nuclei density on parylene-C compared to SiO_2_ surfaces. Error bars represent the standard error (n = 8, 8, 3 and 3 for the PEGylated, FBS immersion, piranha acid and silanized substrates respectively).

The ability of PEGylation to selectively render SiO_2_ cell-repulsive, while leaving the parylene-C cell-adhesive, was assessed by culturing hNT astrocytes on treated substrates for 72 hours and measuring the resulting nuclei density. Figure 1 presents characteristic images of hNT astrocytes cultured on parylene-C/SiO_2_ substrates treated with the PEGylation protocol, the serum-immersion protocol and the intermediary steps of the PEGylation protocol, while Fig. 2 presents data quantifying the cell patterning effect through the nuclei density on each substrate

Figure 1a shows that the PEGylation protocol was successful in preventing astrocyte adhesion without affecting cell adhesion to the parylene-C area of the sample. In contrast, the standard serum immersion protocol resulted in a small amount of cell adhesion to the serum adsorbed SiO_2_ (Fig. 1b). Similarly, while contrast in cell adhesiveness was observed for the two PEGylation control substrates there was a significant amount of cell adhesion to the SiO_2_ regions.

The cut-outs in Fig. 1 show full-resolution images of hNT astrocytes on the respective surfaces. On all parylene-C areas the hNT astrocytes adopted the typical large polygonal morphology that is observed on tissue culture polystyrene^17^. In contrast, hNT astrocytes on the SiO_2_ regions of the serum-immersion substrates were typically smaller in size and extended more astrocytic processes.

Figure 2a presents the mean nuclei density observed on the parylene regions of the treated substrates. A mean nuclei density of 43 ± 4.9 mm^−2^ was observed for the PEGylated substrates, 40 ± 5.2 mm^−2^ for the serum-immersion substrates, 48 ± 9.1 mm^−2^ for the PEGylated substrates without the APTES step and 49 ± 5.8 mm^−2^ for the substrates PEGylated substrates without the m-PEG-SVA step. The differences in the mean nuclei density on the parylene regions were not statistically significant (${H}_{0}:{\mu }_{PC/PEG}=\mu {s}_{PC/FBS}={\mu }_{PC/NoAPTES}={\mu }_{PC/NoPEG},\,p=0.80$).

Figure 2b presents the mean density of astrocytes observed on the SiO_2_ regions of the treated substrates. A mean nuclei density of 1.3 ± 0.42 mm^−2^ was observed on the PEGylated substrates, which was significantly lower than the density of 8.1 ± 1.3 mm^−2^ observed on the serum-immersion substrates, (${H}_{0}:{\mu }_{Si{O}_{2}/PEG}={\mu }_{Si{O}_{2}/FBS},\,p=0.011$), indicating that the PEGylated SiO_2_ was more resistant to cell adhesion than the serum-immersion treated SiO_2_. Similarly, the nuclei density on the PEGylation control substrates without APTES or m-PEG-SVA was 18 ± 4.2 and 10 ± 1.3 mm^−2^ respectively. That nuclei density was also significantly greater than the nuclei density observed for the PEGylated SiO_2_ regions (${H}_{0}:{\mu }_{Si{O}_{2}/PEG}={\mu }_{Si{O}_{2}/NoAPTES},p=1.7\ast {10}^{-04},{H}_{0}:{\mu }_{Si{O}_{2}/PEG}={\mu }_{Si{O}_{2}/NoPEG},p=0.021$).

Figure 2c presents the ratio of the density of nuclei detected on the parylene and SiO_2_ regions of each substrate. A median nuclei ratio of 65 ± 23 was observed for the PEGylated substrates, which was significantly greater than the median ratio of 5.6 ± 0.9 for the FBS immersion treated substrates ($${H}_{0}:{\tilde{x}}_{PEG}={\tilde{x}}_{NoAPTES},\,p=2.5\ast {10}^{-4}$$). Similarly, the median nuclei density ratio for the PEGylated substrates was significantly greater than the PEGylated substrates treated without APTES, 2.7 ± 0.16, and the PEGylated substrates treated without m-PEG-SVA, 4.6 ± 0.02 ($${H}_{0}:{\tilde{x}}_{PEG}={\tilde{x}}_{NoAPTES},\,p=4.2\ast {10}^{-4},\,{H}_{0}:{\tilde{x}}_{PEG}={\tilde{x}}_{NoPEG},\,p=0.0027$$$$)$$

Considered together, the data in Fig. 2 indicate that the decrease in cell adhesion of PEGylated SiO_2_ surfaces can be attributed to attachment of PEG to SiO_2_ regions, rather than the piranha acid or silanization procedures alone. Furthermore, the PEGylation procedure does not affect the cell adhesiveness of parylene-C and results in significantly better contrast in the cell adhesiveness compared to the standard FBS immersion protocol.

### Influence of PEGylation on Astrocyte Isolation

**Figure 3:**
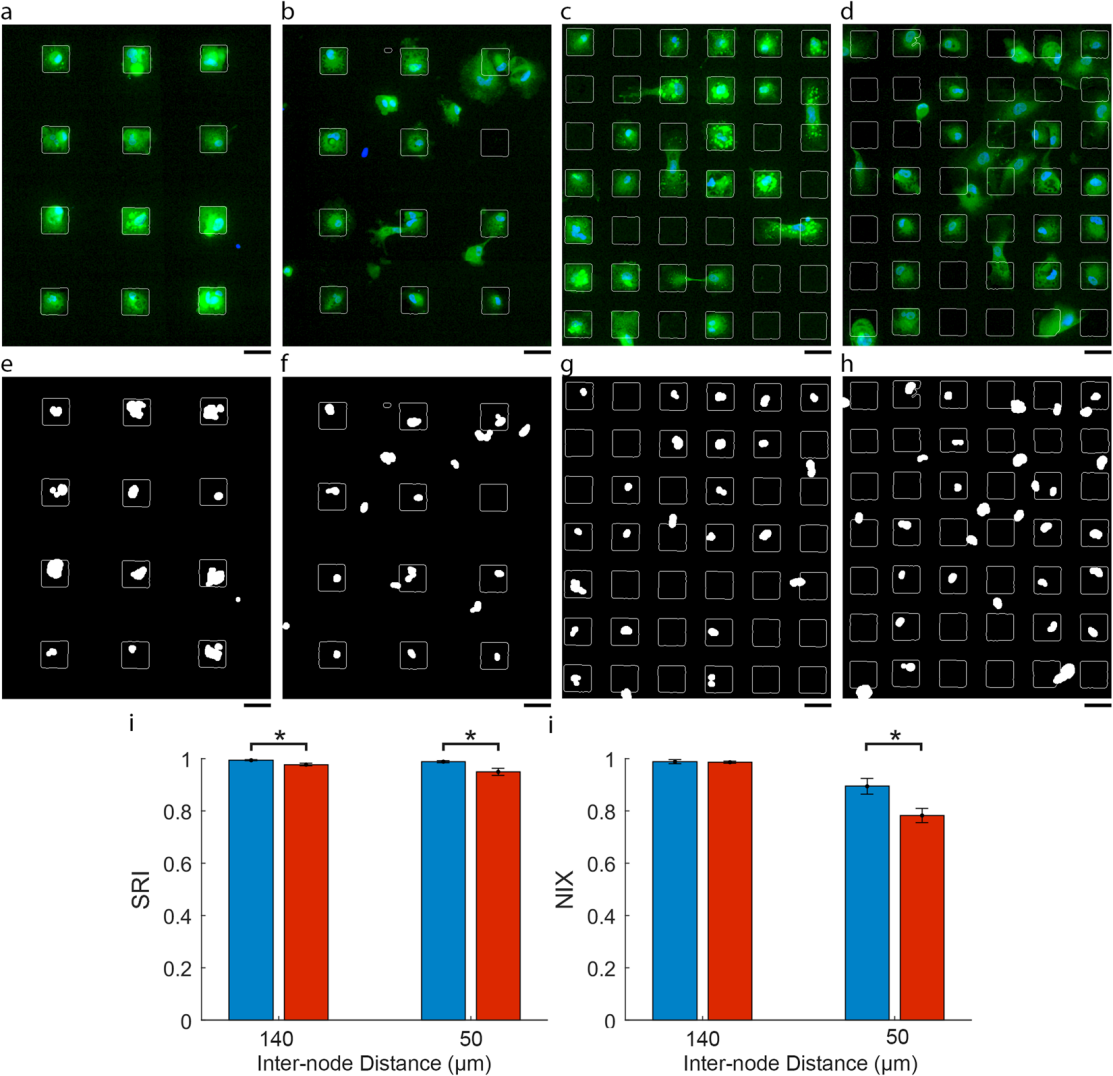
Characterisation of astrocyte patterning quality on PEGylated and FBS immersed parylene-C/SiO_2_ substrates with 50 × 50 µm parylene nodes designed to isolate single cells. Astrocyte cytoplasms were labelled after 72 hours with CMFDA (green) and the nuclei were labelled with Hoechst 33258 (blue). (**a**–**d**) CMFDA and Hoechst 33258 fluorescence images. (**e**–**h**) Thresholded Hoechst 33258 indicating nuclei locations. (**a**,**e**) PEGylated substrates with an inter-node distance of 140 µm. (**b**,**f**) FBS immersion substrates with a inter-node distance of 140 µm. (c, g) PEGylated substrates with an inter-node distance of 50 µm. (**d**,**h**) FBS immersion substrates with a inter-node distance of 50 µm. (**i**) Quantification of Silicon Repulsion Index (SRI). (**j**) Quantification of Node Isolation Index (NIX). Legend: Blue, PEGylation protocol, Red, serum immersion protocol. Scale bars represent 50 µm. Error bars represent the standard error (n = 8). Significant differences are indicated by astericks (P < 0.05). Images were modified by adjusting the contrast only and the parylene regions are outlined in white.

The influence of PEGylation on the quality of cell patterning was further assessed on substrates designed to isolate single astrocytes by comparing the Silicon Repulsion Index (SRI) and the Node Isolation Index (NIX) values relative to serum-immersion controls. The SRI represents the fraction of the SiO_2_ area that is not covered by cellular content and is therefore a measure of how well the surface resists cell adhesion. The NIX represents how well the pattern isolates cells by preventing cells connecting two or more nodes. The NIX was calculated as the inverse of the number of connected nodes. Figure 3a–d show characteristic images of astrocytes cultured on the respective patterned substrates, Fig. 3e–h show processed Hoechst fluorescence images that highlight the isolation of astrocytes and Fig. 3i and j quantify the pattern conformity using the SRI and NIX metrics respectively.

Figure 3a and e demonstrates that, when treated with the PEGylation protocol, parylene nodes at an inter-node distance of 140 µm effectively isolate single astrocytes to individual nodes with little off-parylene cell growth, resulting in a SRI value of 0.99 ± 0.0021. Similarly, Fig. 3b and f shows the equivalent FBS immersion treated substrates isolate single astrocytes to individual nodes, however, more off-parylene cell growth was observed, resulting in a significantly lower SRI value 0.97 ± 0.005 (${H}_{0}:{\mu }_{140\mu m/PEG}={\mu }_{140\mu m/FBS},\,p=0.0054$). While the off-parylene growth observed on the FBS treated substrates resulted in a significant decrease in the SRI, the astrocytes did not tend to contact multiple parylene nodes. As a result there was no significant difference in the NIX values obtained for the PEGylated and FBS immersion treated substrates with respective NIX values of 0.99 ± 0.0017 and 0.99 ± 0.0094. ($NIX-{H}_{0}:{\mu }_{140\mu m/PEG}={\mu }_{140\mu m/FBS},\,p=0.81$).

Figure 3c and g shows PEGylated substrates with the parylene nodes at an inter-node distance of 50 µm. Isolation of single astrocytes was observed and off-parylene astrocyte growth appeared to be limited to single astrocytes occupying two adjacent parylene nodes resulting in a SRI of 0.98 ± 0.004 that was not significantly different from the 140 µm PEG nodes ($SRI-{H}_{0}:{\mu }_{140\mu m/PEG}={\mu }_{50\mu m/PEG},\,p=0.25$). However, the astrocytes that attached to two adjacent nodes by bridging across the PEGylates SiO_2_ surface resulted in a lower NIX value of 0.89 ± 0.030 that was significantly different from the PEG-140 µm nodes ($NIX-{H}_{0}:{\mu }_{140\mu m/PEG}={\mu }_{50\mu m/PEG},\,p=0.0091$). Figure 3d and h shows the equivalent pattern design that had been treated by the serum-immersion protocol. Greater off-parylene growth was observed relative to the PEG resulting in a significantly lower SRI value of 0.95 ± 0.013 ($SRI-{H}_{0}:{\mu }_{50\mu m/PEG}={\mu }_{50\mu m/FBS},\,p=0.025$). Furthermore the off-parylene growth was sufficient to result in a significant decrease in the NIX of 0.78 ± 0.027 ($NIX-{H}_{0}:{\mu }_{50\mu m/PEG}={\mu }_{50\mu m/FBS},\,p=0.019$).

### Validation of Cell Patterning Mechanism

**Figure 4:**
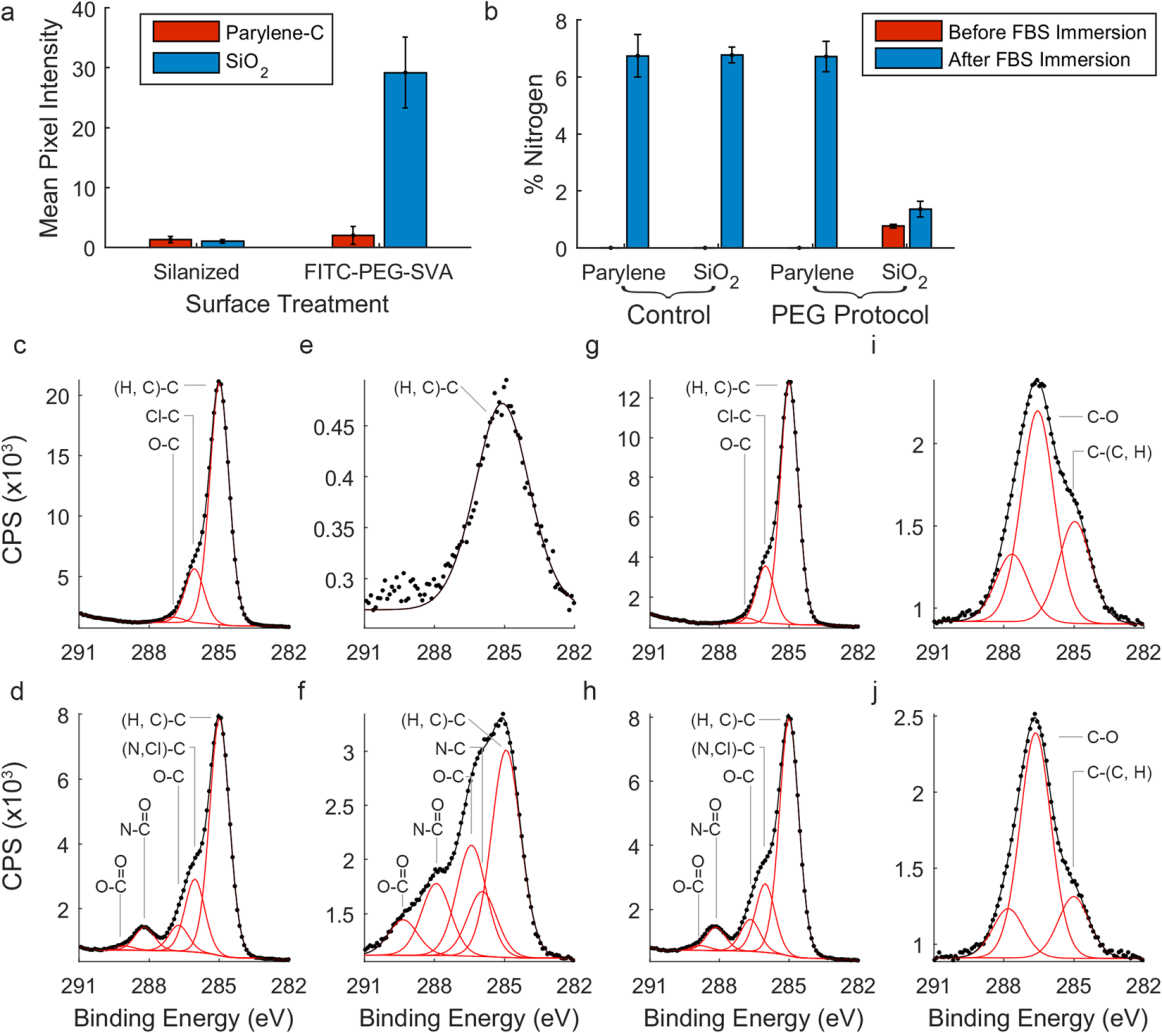
Validation of selective PEGylation and inhibition of protein adsorption to PEGylated SiO_2_ surfaces. (**a**) Mean fluorescence of parylene-C (Red), and SiO_2_ (Blue) surfaces that had been either silanized or PEGylated with fluorescent FITC-PEG-SVA. (**b**) Percentage nitrogen content on control piranha acid treated and PEGylated parylene-C/SiO_2_ substrates before and after incubation in 5% FBS/PBS. (**c**–**j**) C_1s_ XPS spectra, reported as counts per second (CPS), of (**c**,**d**) Piranha acid treated parylene-C, (**e**,**f**) Piranha acid treated SiO_2_, (**g**,**h**) PEGylation treated parylene-C, (**i**,**j**) PEGylation treated SiO_2_. (**c**,**e**,**g**,**i**) show surfaces before serum immersion and (**d**,**f**,**h**,**i**) show surfaces after immersion in 5% FBS/PBS for 1 hour.

Selective PEGylation of SiO_2_ surfaces was verified by PEGylating the substrates with fluorescein conjugated polyethylene glycol succinimidyl valerate (FITC-PEG-SVA). The mean fluorescence intensity was compared between silanized and FITC-PEGylated surfaces. Figure 4a demonstrates that there was no significant change in the mean fluorescence observed between silanized and FITC-PEG treated parylene-C surfaces (1.3 ± 0.3 (s.d.) and 1.5 ± 0.5 (s.d.) respectively). However, the mean fluorescence intensity on SiO_2_ surfaces increased from 2.0 ± 1.5 (s.d.) to 29 ± 5.9 (s.d.) indicating that the PEGylation was both successful and selective to the SiO_2_ surfaces.

Cell patterning on parylene-C/SiO_2_ substrates, using the traditional serum immersion technique, involves pre-incubating the substrates in serum to allow for protein adsorption. However, the astrocyte culture media also contains 5% FBS. If the serum pre-incubation step was removed, as in the PEGylation protocol, it is conceivable that that serum proteins from the culture media could adsorb to the substrate at the same time as cell attachment. We, therefore verified that cell patterning on PEGylated parylene-C/SiO_2_ substrates was due inhibition of protein adsorption on SiO_2_ surfaces by measuring the amount of adsorbed serum protein following a 1 hour incubation in 5% FBS/PBS. Figure 4b–j quantifies the amount of adsorbed protein on PEGylated parylene-C/SiO_2_ substrates before and after serum immersion and compares this with parylene-C/SiO_2_ substrates that have been treated with piranha acid only. Protein adsorption was measured by determining the total nitrogen content on each surface using X-ray photoelectron spectroscopy (XPS).

Figure 4b shows the percentage nitrogen content on parylene-C/SiO_2_ substrates prepared using either the traditional serum-immersion protocol or the PEGylation protocol, both before and after incubation in 5% FBS/PBS for 1 hour. The parylene-C and SiO_2_ surfaces of the control substrates showed 0% nitrogen before serum immersion and 6.7% nitrogen following serum immersion. Similarly the parylene-C surface of PEGylated substrates showed 0% nitrogen before serum immersion and 6.7% nitrogen following serum immersion. Significantly, the nitrogen content on PEGylated-SiO_2_ surfaces was reduced to 1.3% following serum immersion. However, the PEGylated SiO_2_ surfaces also contained 0.76% nitrogen before serum incubation. This can be accounted for by nitrogen in the amide bond that covalently bonds the silanized SiO_2_ to PEG. The relative increase in nitrogen content on PEGylated SiO_2_ surfaces was therefore 0.54% percentage points.

Finally, Fig. 4c–j show C_1s_ XPS spectra for each of the surfaces reported in Fig. 4b. The presence of adsorbed protein can be inferred by the C_1s_ peak at 288.2 eV, which is attributed to carbon present in amide bonds in protein^18–20^. Figure 4c and d show parylene-C surfaces, treated with the serum-immersion protocol, before and after serum incubation respectively. Similarly Fig. 4e and f SiO_2_ surfaces, treated with the serum-immersion protocol, before and after serum immersion. Both serum-immersion treated parylene-C and SiO_2_ surfaces show the presence of the amide C_1s_ peak at 288.2 eV, indicating the presence of adsorbed protein.

Figure 4g and h show parylene-C surfaces, treated with the PEGylation protocol, before and after serum immersion respectively. Similarly, Fig. 4i and j show SiO_2_ surfaces treated with the PEGylation protocol before and after serum immersion respectively. The presence of the amide C_1s_ peak at 288.2 eV on parylene-C treated with the PEGylation protocol indicates that PEGylation does not affect protein adsorption the parylene-C surface. However, comparing Fig. 4i and j, little change is observed in the shape of the C_1s_ spectrum indicating minimal protein adsorption to PEGylated SiO_2_ surfaces.

## Discussion

In this work, we have demonstrated a simple, superior alternative activation protocol to enable cell high-fidelity patterning on the parylene-C/SiO_2_ platform. Previously, in order to enable patterning, the substrates needed to be immersed in serum. During this time serum proteins differentially adsorbed to the parylene-C and SiO_2_ regions, rendering the parylene-C cell-attractive and the SiO_2_ cell-repulsive. However, the batch to batch inconsistencies typically associated with serum render this method undesirable, in addition to the fact that the patterning never achieved 100%. In this work, we have demonstrated that PEGylation provided greater contrast in cell adhesiveness achieving a median ratio of the nuclei density of 65:1 compared to 5.6:1 for the standard serum-immersion protocol.

In our previous work we demonstrated the ability to isolate single astrocytes using the parylene-C/SiO_2_ platform^12,17^. Astrocytes were observed to localise on individual parylene nodes, with their cytoplasm entirely constrained by the extent of the parylene regions. However, in many cases we observed the cytoplasm would grow into the surrounding SiO_2_ areas. Therefore, in order to maintain cell isolation it was necessary to space adjacent parylene nodes sufficiently apart. Delivopoulos *et al*. observed a similar effect when culturing rodent glia on parylene strips in long term culture^21^. The astrocytes cytoplasm remained confined to the parylene strips for up to 7 days *in vitro* (DIV), however after 21 DIV conformity to the pattern was significantly degraded. Delivopoulos *et al*. reported that, as the total protein content on the SiO_2_ and parylene-C areas remained relatively constant over the course of the experiments, the loss of conformity was most likely due to glial cell division.

Currently, with the parylene-C SiO_2_ platform high fidelity patterning is limited by the fact that astrocytes exhibit a small tolerance for growing on the cell repulsive SiO_2_ regions. The fidelity of patterning is therefore a function of the underlying pattern geometry as the parylene regions need to be spaced far enough apart to prevent the cytoplasm of neighbouring astrocytes from touching. In this work, we have demonstrated that PEGylation of the SiO_2_ regions can improve the contrast in cell adhesion thereby reducing the limitations on the geometry of the underlying pattern.

Using the standard serum immersion activation protocol parylene nodes spaced at 140 µm, in order to compensate for off-parylene growth, achieved a SRI of 0.97. When the node spacing was reduced to 50 µm SRI decreased further to 0.95. While the difference in SRI is small, but still statistically significant, the corresponding decrease in the NIX is much greater, reducing from 0.98 to 0.78.

The same dependence on node spacing was, however, not observed with PEGylated substrates. Using the PEGylation protocol parylene nodes spaced at 140 µm achieved a SRI of 0.99. When the node spacing was reduced to 50 µm SRI remained the same at 0.99 and in both cases this was significantly greater than the SRIs obtained with the FBS immersion protocol. While the NIX also decreased from 0.98 to 0.90 the decrease was less than for the serum-immersed substrates. Furthermore, a qualitative comparison of Fig. 3c and d suggests that the decrease in the NIX associated with PEGylation can be attributed to single astrocytes occupying two adjacent nodes without spreading their cytoplasm to the neighbouring SiO_2_ region. In contrast, the decrease in the NIX associated with serum immersion results from astrocytes that attach and spread their cytoplasm onto the SiO_2_ areas, thereby making contact with neighbouring cells.

Therefore, not only does replacing the serum-immersion activation protocol with the PEGylation protocol mitigate the drawbacks of relying on poorly defined serum, it also improves the fidelity of glial patterning.

Finally, while PEGylation typically requires careful selection of appropriate surface chemistries the appropriate surface chemistry is innate to the parylene-C/SiO_2_ platform as pure parylene-C does not contain free hydroxyl groups. It has been reported by Delivopoulos *et al*. that untreated parylene-C, deposited by chemical vapor deposition, contains approximately 6% atomic oxygen at the parylene surface^22^. Furthermore, parylene-C is susceptible to oxidation by UV light causing scission of the methylene groups and the formation of hydroxyl, aldehyde and carboxyl groups^23^. UV exposure can increase surface oxygen concentrations to approximately 25%^22^. We report that the surface of our parylene-C substrates contained approximately 2% oxygen after piranha acid treatment, determined by x-ray photoelectron spectroscopy, however, Fig. 4a demonstrates this does not appear to result in significant PEGylation of the parylene-C surface and therefore did not affect astrocyte adhesion to parylene-C. Similarly Fig. 4b demonstrates that protein adsorption was unaffected on parylene-C regions that had been treated with the PEGylation protocol whereas the PEGylated SiO_2_ regions resisted protein adsorption.

## Methods and Materials

### Fabrication and Design of Parylene-C/SiO2 Substrates

Parylene-C/SiO_2_ substrates were prepared using a femtosecond laser ablation protocol that has previously been described in detail by Raos *et al*.^12^. The protocol is briefly outlined below. First, silicon wafers were thermally passivated to form a 200 nm layer of SiO_2_. A 100 nm thick layer of parylene-C was then deposited on top of the SiO_2_ by chemical vapor deposition (Speciality Coating Systems). The wafers were then diced into 7 × 7 mm squares. Next, the output of a Coherent Legend femtosecond pulsed laser (800 nm, 100 fs pulses, repetition rate 500 Hz) was transmitted through a chrome-plated quartz crystal mask and focussed on the surface of the parylene-C. A computer-controlled motorized stage moved the substrate relative to the beam to ablate parylene-C from pre-defined regions.

**Figure 5:**
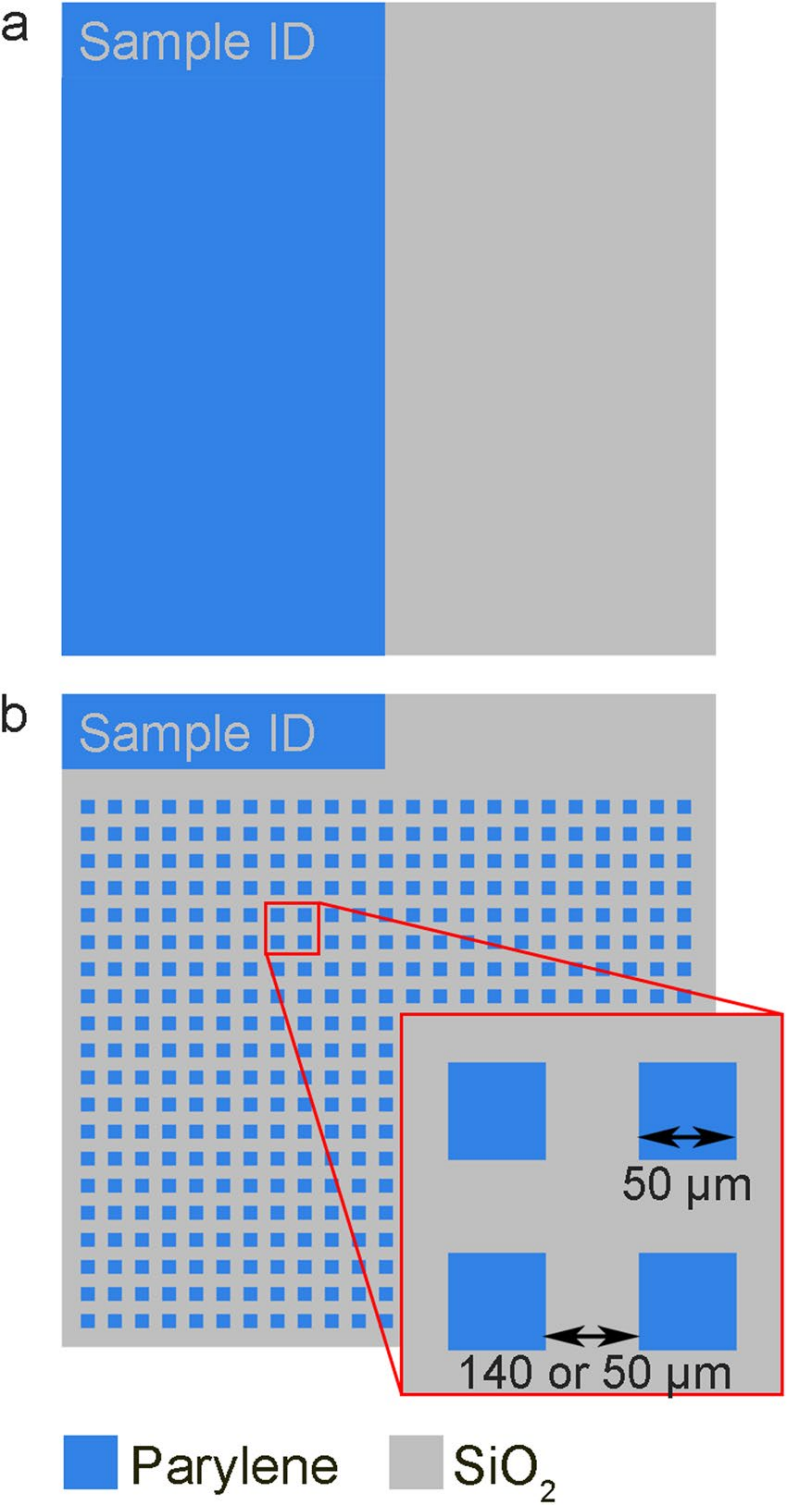
Design of parylene-C/SiO_2_ substrates used to assess the quality of glial patterning. (**a**) Design to assess overall cell adhesiveness to parylene-C and SiO_2_ regions. (**b**) Design to assess isolation of single astrocytes.

Previous work has demonstrated that, although hNT astrocytes tend to pattern on parylene nodes, the cytoplasm can show tolerance for the normally repulsive serum adsorbed SiO_2_, reducing cell isolation^17^. Therefore, for this work we assessed the quality of cell patterning using two pattern designs. First, we assessed the bulk cell adhesiveness of parylene-C and PEGylated SiO_2_ using the pattern shown in Fig. 5a. Second we assessed the influence of PEGylation of substrates designed to isolate single astrocytes, in close proximity to one another, shown in Fig. 5b. These substrates consisted of 50 × 50 µm square parylene nodes with and inter node spacing of either 140 µm or 50 µm. A spacing of 140 µm has previously been shown to be sufficiently large to prevent neighbouring astrocytes from touching if the cells are not contained by the parylene nodes^17^, whereas 50 µm is approximately 3 times smaller than the typical size of the hNT astrocyte cytoplasm and allows connections between cells in the case of poor patterning contrast.

### PEGylation of Parylene-C/SiO2 Substrates

**Figure 6:**
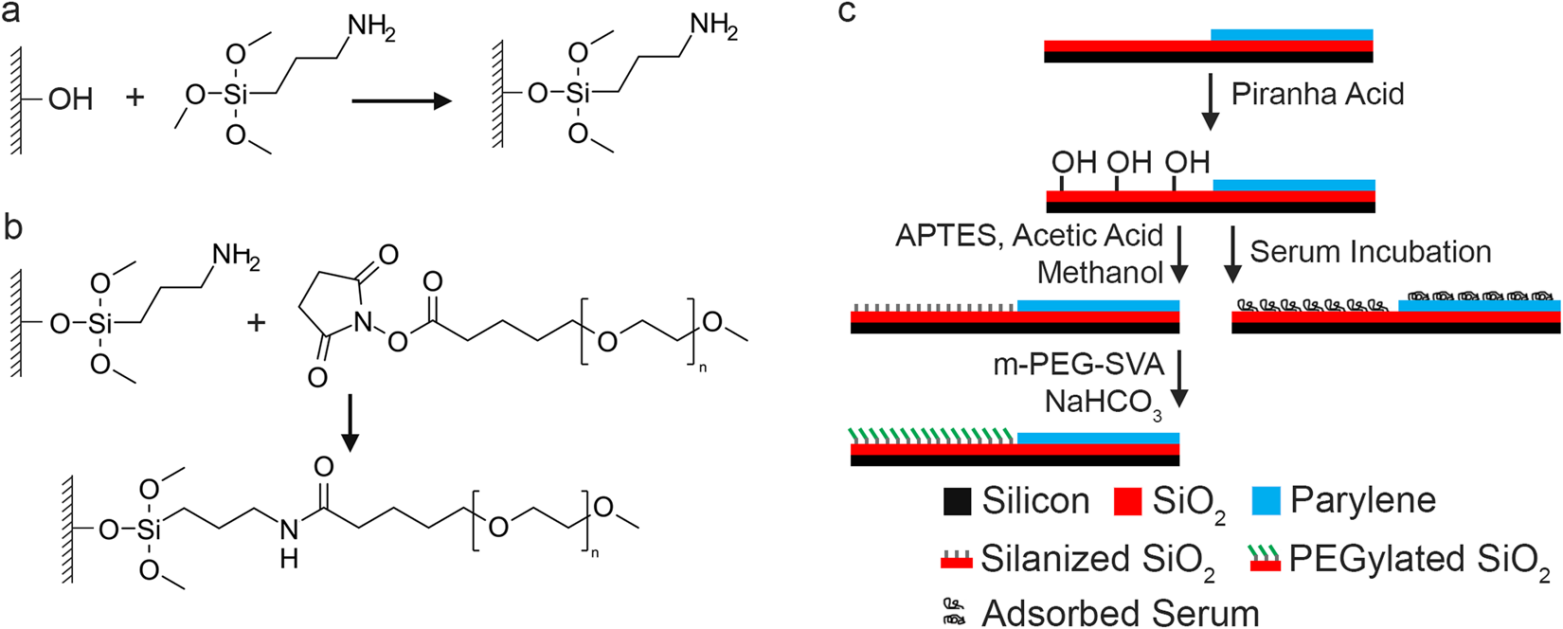
(**a**) Reaction of amino-silane with free hydroxyl functional group. (**b**) Reaction of succinimidyl valerate-PEG with surface bound amino-silane. (**b**) Schematics of the PEGylation protocol (left) and the standard serum immersion protocol (right).

Selective PEGylation of the SiO_2_ regions on patterned parylene-C/SiO_2_ substrates was performed using a protocol consisting of three main processes – cleaning, silanization, and PEGylation. Substrates were first cleaned in acetone (3 × 10 mL) followed by Milli-Q water (3 × 10 mL) to remove any soluble contamination from their surface. Next, substrates were immersed in a piranha acid solution, made with a 5:3 ratio of 30% hydrogen peroxide to 98% concentrated sulfuric acid. The piranha immersion served a dual purpose. First the piranha acid removed organic residues and debris that may have been introduced onto the surface of the patterned substrates during fabrication. Second, the piranha acid activates the SiO_2_ surface towards silanization by introducing free hydroxyl groups to the surface of the SiO_2_ (Fig. 6a). The free hydroxyl groups represent the chemical functionality required for covalent bonding of an aminosilane molecule to the SiO_2_ surface (Fig. 6b). Pure parylene-C does not contain free hydroxyl functional group and is therefore resistant to silanization. The PEGylation protocol is summarised in Fig. 6c.

Following the piranha acid clean the substrates were rinsed in Milli-Q water (5 × 10 mL), followed by methanol (3 × 10 mL). The substrates were then transferred to a silanization solution consisting of methanol (20 mL), acetic acid (1 mL) and 3-aminopropyl trimethoxysilane (2 mL, Cat# 281778 Sigma-Aldrich) for 20 min. The substrates were rinsed in methanol (3 × 10 mL) and blown dry with nitrogen.

The PEGylation solution was prepared by dissolving 15 mg of methyl-polyethylene glycol succinimidyl valerate (MW 5000, Laysan Bio Inc., Cat# MPEG-SVA-5000) in 150 µL of sodium bicarbonate buffer (0.1 M, pH 8.5). The solution was mixed and centrifuged for 1 min to remove any bubbles. Next, 10 µL of the PEGylation solution was placed on a clean glass coverslip for each sample to be PEGylated and the substrates were placed face down on the in the PEGylation solution. The reaction was then left to occur at room temperature in a dark humid environment for approximately 18 hours. PEGylated substrates were then rinsed thoroughly in sterile Milli-Q water to remove any non-bound PEG, blown dry with nitrogen, and used immediately for cell culture.

The success of the PEGylation reaction was verified through the use fluorescein conjugated polyethylene glycol succinimidyl valerate (FITC-PEG-SVA, MW 5000, NanoCS, Cat# PG2-FCNS-5k). The PEGylation protocol was performed as described, with the substitution of FITC-PEG-SVA. FITC-PEGylated parylene-C/SiO_2_ substrates were imaged for 1 s on an Olympus BX53 using an 8-bit XC50 camera, mercury bulb illumination and GFP filter set (470–495/550 nm, Em/Ex) to quantify the relative levels of PEGylation.

Furthermore, in order to ensure cell patterning could be attributed to PEGylation two sets of control substrates were made. First, the protocol was performed as described above but without APTES in the silanization mixture, in order to attribute cell patterning to silanization and subsequent PEGylation, rather than the piranha acid treatment. Second, the protocol was performed as above but without mPEG-SVA in the PEGylation mixture, in order to attribute cell patterning to the presence of PEG rather than silanization.

Finally, substrates were prepared using the standard serum incubation protocol originally described by Delivopoulos *et al*.^1^. Briefly, substrates were cleaned in acetone, Milli-Q water, and piranha acid and then rinsed in Milli-Q water as described above. Substrates were then immersed in 500 µL FBS each for an incubated 3 hours at 37 °C. Following incubation, the substrates were gently rinsed with PBS and used immediately for cell culture.

### hNT Astrocyte Differentiation Protocol and Cell Seeding

hNT astrocytes were differentiated from the NTera2.D1 (NT2/hNT) cell line (ATCC) using the protocol described in Unsworth *et al*.^17^. Briefly, NT2 cells were plated in 10% FBS/DMEM:F12 in Petri dishes at 6 × 10^6^ cells per dish and treated with retinoic acid (RA) at 10 µM for 2 weeks, replating every 2–3 days. Cells were then transferred to T75 flasks and treated with RA for a further 7–10 days with media changes every 2–3 days (10% FBS/DMEM:F12). Cells with neuronal morphology were removed by selective trypsinization and the remaining non-neuronal cells were replated into uncoated T75 flasks. Cells were then treated in 5% FBS/DMEM:F12 with decreasing concentrations of mitotic inhibitors uridine (Urd) 10 µM, 5-fluoro-2′-deoxyuridine (FUdR) 10 µM and $\beta$-D-arabinofuranoside (AraC) 1 µM for 12 days, Urd and FUdR (10 µM) for 13 days followed by Urd (10 µM) for 8 days, with media changes every 2–3 days. The astrocytes were harvested by trypsinization for seeding onto patterned parylene/SiO_2_ substrates. hNT astrocytes were seeded in 200 µL aliquots onto the samples at 50 cells per mm^2^.

### Fluorescence Labelling and Imaging

The quality of cell patterning was assessed 72 hours following cell seeding. Prior to fixation (3.6% PFA for 10 min at ambient conditions), astrocyte cytoplasms were labelled with CMFDA (1 µM, 1 hour at 37 °C and 5% CO_2_), and astrocyte nuclei were labelled with Hoechst 33258 (1.6 µg mL^−1^, 20 min at 37 °C and 5% CO_2_). All imaging was performed on an Olympus BX53 using an 8-bit XC50 camera, mercury bulb illumination, 10xobjective (UMPLFLN, Olympus) and GFP and DAPI filter sets (470–495/550 nm and 360–370/410, Em/Ex).

### Image Acquisition and Processing

Custom software written in Matlab© (2014b, The MathWorks Inc., Natick, MA) was used for image processing. Histogram equalization was applied to the images to equally distribute the data across the 8-bit dynamic range, followed by a 5 × 5 Wiener filter to reduce noise in the images. Fluorescence images were binarized and objects in the masks representing noise were subsequently removed by morphological opening using a 7-pixel disk structural element. The masks representing parylene, cellular and nuclear areas were further processed to generate metrics quantifying the level of patterning on each substrate.

Cell patterning was initially quantified by calculating the nuclei density from the nuclear and parylene masks. Next the Silicon repulsion index (SRI) was calculated, as used by Hughes *et al*.^9^. The SRI represents the total SiO_2_ area that is not covered by cellular content, i.e. objects in the cellular mask image. A SRI value of 1 represents complete lack of cell adhesion to the SiO_2_ areas, while a SRI value of 0 represents complete coverage by cellular content. For this work, we also introduce a new metric to quantify the quality of patterning in our cultures, the Node Isolation Index (NIX). The Isolation Index represents the isolation of a cluster of cells, on a given parylene node, from cells on adjacent parylene nodes. The NIX is calculated by first determining, for each parylene node, the number of nodes that are connected to it by cellular material (CMFDA fluorescent objects). The inverse of the number of connected nodes represents the NIX, which is then averaged over every node on the sample. Consequently the NIX can take values approaching 0, representing poor isolation, where a node is connected to many other nodes, to 1, representing perfect isolation, where no two nodes are connected by an astrocyte.

### Protein Adsorption Analysis with X-Ray Photoelectron Spectroscopy

Parylene-C/SiO_2_ substrates were treated with either piranha acid or the PEGylation protocol and immersed in 5% FBS/PBS for 1 hour. Samples were then individually rinsed in Milli-Q water (3 × 10 mL) and allowed to dry. XPS spectra were recorded on a Kratos Axis Ultra DLD spectrometer (Kratos Analytical, Manchester UK). Survey spectra and Carbon 1 s (C_1s_) elemental spectra were collected from 300 × 700 µm areas on the parylene-C and SiO_2_ regions of each sample. Survey spectra were collected with a pass energy of 160 eV and dwell time of 138 ms from five 180 s sweeps. Elemental spectra were collected with a pass energy of 20 eV and a dwell time of 260 ms from fifteen 60 seconds sweeps. The vacuum chamber pressure was kept below 2 × 10^−9^ Torr. Samples were illuminated with monochromated Aluminium ${K}_{\alpha }$ X-rays at 1486.69 eV and analysed with charge neutralisation. Analysis of the XPS data was performed in CasaXPS. C_1s_ spectra were calibrated to the C-C peak at 285 eV and fitted with a Shirley type background. Peaks were deconvolved using Gaussian/Lorentzian (70%/30%) line shape and constrained to have the same FWHM. C_1s_ peak assignments were made by referencing the work of Das *et al*.^18^, Gruian *et al*.^19^, Ray *et al*.^20^, Golda *et al*.^24^, Santucci *et al*.^25^ and Sharma *et al*.^26^. Spectra intensity is presented as electron counts per seconds (CPS).

### Statistical analysis

Statistical analysis of the patterned cultures was performed in MATLAB using the Statistics Toolbox. The datasets generated during and/or analysed during the current study are available from the corresponding author on reasonable request. For data representing the ratio of nuclei densities it was necessary to log-transform the data to ensure equality of variance, and consequently these data are reported as median values, otherwise the data is reposted as mean values. Null hypotheses and p-value are reported in the text where relevant. A p-value of less than 0.05 was considered significant. Unless otherwise stated the symbol ± refers to the standard error of means. One-way ANOVA with a Bonferroni correction was used to compare between treatments. We adopt the notation ${\mu }_{{Substrate}/{Treatment}}$ to refer to the average value of the metrics used to quantify patterning on both substrates with various treatments. *µ*_*PC*_ refers to parylene-C surfaces, *µ*_*SiO2*_ refers to silicon dioxide surfaces, *µ*_*PEG*_ refers to substrates treated with the PEGylation protocol, *µ*_*FBS*_ refers to the standard FBS immersion protocol, *µ*_*No APTES*_ refers to the PEGylation protocol excluding the APTES treatment, *µ*_*No PEG*_ refers to the PEGylation protocol excluding the mPEG-SVA treatment, *µ*_*140µm*_ refers to substrates with 140 µm parylene nodes and *µ*_*50µm*_ refers to substrates with 50 µm parylene nodes.

## Conclusion

In this work, we have demonstrated how the innate contrast in surface chemistry on the parylene-C/SiO_2_ cell platform enable selective PEGylation of SiO_2_ regions for improved glial cell patterning. PEGylated substrates enable greater contrast in astrocyte adhesion compared to the standard serum immersion protocol. Furthermore, greater astrocyte isolation was achieved on PEGylated substrates. This alternative patterning protocol mitigates the uncertainty in the composition of serum from different batches.
